# Genetic and Functional Changes in Mitochondria in the Pituitary Adenoma: The Pathogenesis and Its Therapy

**DOI:** 10.3390/antiox13121514

**Published:** 2024-12-11

**Authors:** Hansen Wu, Jie Xu, Wenxuan Zhao, Weiqiang Lv, Zhihui Feng, Lijun Heng

**Affiliations:** 1 Frontier Institute of Science and Technology, Xi’an Jiaotong University, Xi’an 710049, China; whs740211756@outlook.com (H.W.); 1910990771@stu.xjtu.edu.cn (W.Z.); 2 Center for Mitochondrial Biology and Medicine, The Key Laboratory of Biomedical Information Engineering of Ministry of Education, School of Life Science and Technology, Xi’an Jiaotong University, Xi’an 710049, China; xj0322@mail.xjtu.edu.cn (J.X.); gl824705609@stu.xjtu.edu.cn (W.L.); 3 School of Health and Life Sciences, University of Health and Rehabilitation Sciences, Qingdao 266071, China; 4 Department of Neurosurgery, Tangdu Hospital, Air Force Medical University, Xi’an 710038, China

**Keywords:** pituitary adenomas, mitochondrial dysfunction, oxidative stress, natural compounds

## Abstract

Pituitary adenoma is a common neoplasm of the pituitary gland. Although most pituitary adenomas are benign, they can pose significant challenges in terms of their consequences and prognosis due to their tendency to invade surrounding tissues and their effects on hormone secretion. The management of pituitary adenomas typically involves surgery, medical therapy, and radiotherapy, each of which has its own limitations. Mitochondria play a crucial role in tumor development and progression by regulating various metabolic processes and signaling pathways within tumor cells and the tumor microenvironment. Multiple studies have indicated that mitochondrial dysfunction is implicated in human pituitary adenomas. Furthermore, several compounds with therapeutic effects on pituitary adenomas have been reported to target mitochondrial function. In this review, we summarize recent studies that highlight the involvement of mitochondrial homeostasis imbalance in the biology of pituitary adenomas. We conclude that mitochondria may represent a promising therapeutic target for the treatment of pituitary adenomas.

## 1. Introduction

Pituitary adenomas are neoplasms originating from the adenohypophyseal cell lineage of the pituitary gland. As one of the most common types of intracranial tumors, pituitary adenomas account for 10% to 15% of all intracranial masses. Approximately 70% of these tumors are functional adenomas, which produce excess hormones, including prolactinomas, somatotropinomas, and cortcotropinomas [1]. Patients diagnosed with pituitary adenomas often exhibit complex clinical manifestations [2], primarily characterized by pathological and physical changes resulting from hormonal imbalances, such as those seen in acromegaly induced by somatotropinomas [3]. Pituitary adenomas are usually benign tumors, and the clinical outcomes for patients can be improved by combining various treatment modalities, including surgery [4], radiation therapy [5], and pharmacologic interventions [6]. However, ongoing research has revealed a growing number of complications associated with these tumors, including the development of depression [7]. Additionally, the damage and nerve compression caused by invasive tumors and macroadenomas (≥10 mm) cannot be overlooked [8]. Pituitary adenomas that are highly invasive or too large for surgical intervention present significant challenges in clinical treatment [9]. Moreover, the high recurrence rate of pituitary adenomas, coupled with the need for long-term postoperative monitoring [10], has significantly increased the costs associated with their management. This situation not only inflicts considerable physical and emotional distress on patients but also underscores the gaps in our understanding of pituitary adenomas.

Mitochondria are involved in various cellular processes, including adenosine triphosphate (ATP) generation, calcium sequestration, and reactive oxygen species (ROS) production. They play a crucial role in regulating cellular energy, apoptosis, and autophagy [11]. Mitochondrial dynamics’ characteristics of fusion and division are vital for maintaining basic functions and overall cellular health [12]. Mitochondrial dysfunction is also a hallmark of aging, often marked by irregular mitochondrial morphology, insufficient ATP production, and increased ROS levels [13]. Mitochondrial homeostasis is regulated by both nuclear and mitochondrial DNA (mtDNA) [14]. Abnormal mtDNA expression induced by mitochondrial epigenetics or certain pathological processes can disrupt mitochondrial function, leading to impaired dynamics and quality control [15,16]. Consequently, mitochondrial dysfunction is a common feature in the pathophysiology of various diseases. For instance, Wei et al. highlighted the role of mitochondrial dysfunction in diabetic kidney disease [17], while Johnson et al. found an association between mitochondrial dysfunction and neurodegenerative diseases [18]. Additionally, Wang et al. discovered the involvement of mitochondria in dilated cardiomyopathy [19].

Metabolic reprogramming is one of the hallmarks of cancer [20], making it clear that mitochondria are essential for tumor growth. Recent studies support the consensus that mitochondria play a crucial role in tumor progression, emphasizing both the intrinsic and extrinsic mechanisms through which they exert their influence [21]. Furthermore, the crosstalk between macrophages and cancer cells in the tumor microenvironment involves mitochondrial transfer, which can also regulate cancer progression [22]. The relationship between mitochondria and pituitary adenomas has garnered significant attention in recent research. By investigating the role of mitochondria in energy metabolism, autophagy, immune response, and targeted therapy [23,24], we can deepen our understanding of the pathogenesis of pituitary adenomas and pave the way for new diagnostic and therapeutic strategies.

Focusing on mitochondria as a foundation for treating pituitary adenomas represents an emerging field of research that seeks to elucidate the mechanisms underlying these tumors through mitochondrial function. This approach offers promising opportunities for developing innovative immunotherapies and treatment strategies by targeting mitochondrial dynamics, metabolism, and overall function. In summary, the interplay between mitochondria and tumors is complex, involving intricate metabolic processes, signaling pathways, and interactions within the tumor microenvironment. Continued research in this area is likely to yield new insights for the treatment and prognosis of pituitary adenomas.

## 2. Mitochondria Genetic Disorder in Pituitary Adenomas

The advancement of gene sequencing technology has significantly enhanced our understanding of the relationship between specific gene mutations and the occurrence and progression of tumors. It is now recognized that approximately 5% of hereditary pituitary adenomas are linked to identifiable mutations [25]. In 1990, Landis et al. were the first to employ polymerase chain reaction and allele-specific oligonucleotide hybridization to identify activating mutations in growth hormone-secreting tumors [26]. The maternally expressed gene 3 (MEG3) protein, derived from the long non-coding RNA *MEG3*, is markedly downregulated in various tumor tissues and cell lines, impacting tumor progression. Subsequent research has established a connection between the *MEG3* gene and the pathogenesis of pituitary adenomas [27]. Cyclin-dependent kinase inhibitor 1B (CDKN1B), also known as p27Kip1, is a crucial cell cycle regulatory protein that plays a significant role in cell cycle control; the mutations in the *CDKN1B/p27Kip1* gene are reported to be associated with pituitary adenomas [28]. The aromatic hydrocarbon receptor-interacting protein (AIP) has been implicated in familial isolated pituitary adenoma (FIPA). Daly et al. found that pituitary adenomas resulting from *AIP* mutations are predominantly growth hormone-secreting tumors, although other types of pituitary adenomas have also been observed [29]. Conversely, the multiple endocrine neoplasia type 1 (*MEN1*) gene, a tumor suppressor gene associated with multiple endocrine neoplasia type 1, is rarely mutated in sporadic pituitary adenomas [30]. This highlights the complexity of genetic factors in the pathogenesis of these tumors.

Mitochondria, as cellular energy hubs, play a crucial role in two essential metabolic pathways required for ATP production: the tricarboxylic acid (TCA) cycle, which generates cellular energy and biosynthetic precursors, and oxidative phosphorylation, which operates through the electron transport chain. Succinate dehydrogenase (SDH) is the only mitochondrial protein complex that links the TCA cycle to the electron transport chain, positioning it centrally in cellular energy metabolism [31]. SDH consists of four nuclear-encoded subunits: succinate dehydrogenase A (*SDHA*), succinate dehydrogenase B (*SDHB*), succinate dehydrogenase C (*SDHC*), and succinate dehydrogenase D (*SDHD*). Among these, *SDHA* and *SDHB* function as the catalytic subunits, while *SDHC* and *SDHD* anchor the complex to the mitochondrial inner membrane and provide binding sites for ubiquinone. Dysfunction in *SDH* subunits is associated with various pathological conditions. For instance, mutations in *SDH* can lead to the development of pituitary adenomas [32]. Research using *Sdhb+/−* mice has shown that pituitary hyperplasia in *SDHx*-deficient cells may trigger the formation of these adenomas [33]. Furthermore, extensive genome sequencing and analysis of numerous clinical samples have revealed a strong correlation between germline mutations in the *SDHB* gene and the occurrence of malignant tumors, alongside poor prognoses [34]. Similarly, mutations in the *SDHD* gene are frequently observed in invasive pituitary adenomas [35]. In addition to mutations in the *SDH* family of genes, alterations in other genes, such as isocitrate dehydrogenase (*IDH*) and heat shock 60 kD protein 1 (*HSPD1*) [36,37], have also been detected through sequencing. Table 1 summarizes the mitochondrial gene mutations identified in pituitary adenomas and the associated disease types. Beyond mutations in genes belonging to the mitochondrial proteome, the genetic landscape of pituitary adenomas has been thoroughly investigated. For example, Daly et al. found that approximately 15% of families with FIPA harbor germline mutations in the *AIP* gene [29], which are linked to larger adenomas and younger patient presentations. Identifying both germline and somatic mutations enhances our understanding of the development of pituitary adenomas.

Recent research has increasingly focused on elucidating the gene mutations linked to pituitary adenomas, particularly the role of *SDH* mutations in the development of these tumors. Dwight et al. set out to investigate whether *SDHA* mutations are correlated with pituitary adenoma formation [38]. They identified *SDHA* mutations (c.1873C>T, p.His625Tyr) in a patient with a non-functional pituitary macroadenoma, marking the first report of an association between *SDHA* gene mutations and pituitary adenomas. Their findings suggest that *SDHA* mutations result in the loss of *SDHA* protein expression, subsequently initiating tumor development. In a distinct case, Alzahrani et al. described a highly invasive, aggressive, and drug-resistant giant prolactinoma attributed to pathogenic variations in the *SDHB* (c.343C>T, p.Arg115*) [39]. Guerrero-Pérez et al. [40] identified three types of pathogenic mutations in pituitary adenomas associated with pheochromocytoma/paraganglioma: two mutations in *SDHB* (c.166-170delCCTCA). For patients with *SDH*-deficient tumors but negative genetic test results, intron variations beyond the scope of standard gene sequencing analysis should be considered. De Sousa et al. [41] discovered a novel intronic variation (c.20+74A>G) in the *SDHC* gene, situated within a conserved region of intron 1, which had not been previously documented. Xekouki et al. [33] explored the relationship between *SDH* mutations and both sporadic and familial pituitary adenomas. They identified missense mutations in *SDHB* (c.487T>C, p.Ser163Pro) and *SDHD* (c.149A>G, p.His50Arg; c.53C>T, p.Ala18Val) among patients with sporadic pituitary adenomas. In familial cases, the researchers found a known pathogenic mutation in *SDHD* exon 3 (c.242C>T, p.Pro81Leu). Their analysis suggests that mutations in the *SDHx* gene are associated with the occurrence and progression of pituitary adenomas, particularly growth hormone (GH)—or prolactin—secreting macroadenomas in familial cases. Additionally, a recent study revealed a novel *SDHD* mutation (c.298-301delACTC) that results in premature termination of the protein at position 133 [42], establishing, for the first time, a link between *SDHD* gene mutations and hereditary pheochromocytoma/paraganglioma as well as GH-secreting pituitary adenomas. Table 1 summarizes the various *SDH* mutations identified in pituitary adenomas, highlighting their potential implications for diagnosis and treatment in affected patients. The pituitary adenoma related to *SDH* mutation shows heterogeneous phenotype spectrum. Prolactinoma, growth hormone-secreting adenoma, and non-functional adenoma have all been reported. Although *SDH* mutations can lead to the development of pituitary adenomas, the probability of pituitary adenomas being caused solely by *SDH* mutations is relatively low [43]. In summary, *SDH* mutations are associated with pituitary adenomas, indicating their potential role in tumorigenesis. However, more research is needed to fully understand the impact of these mutations on the development of pituitary adenomas and whether they are fully pathogenic. Genetic testing and molecular research are crucial for further elucidating the relationship between *SDH* mutations and pituitary adenomas.

## 3. Mitochondrial Homeostasis in Pituitary Adenomas

### 3.1. Mitochondria Signaling Pathways in the Pathogenesis of Pituitary Adenomas

Dysregulation of mitochondrial signaling pathways can lead to abnormal cell growth and tumor formation. Low et al. [44] reported on the role of fibroblast growth factor receptor 4 (FGFR4) in the pathogenesis of pituitary adenomas, identifying a novel FGFR4 subtype associated with these tumors. In transgenic mice, expression of a truncated FGFR4 receptor cDNA resulted in the development of lactotroph pituitary adenomas, suggesting that FGFR4 dysregulation may be a key factor in the formation of pituitary adenomas. Similarly, proteins involved in apoptosis, such as apoptotic protease activating factor-1 (APAF-1) and tissue protease B, are linked to both intrinsic and extrinsic mitochondrial pathways. Tanase et al. [45] found that in invasive pituitary adenomas, APAF-1 was significantly downregulated and negatively correlated with invasiveness, while tissue protease B showed a positive correlation with invasiveness. HSPD1, a member of the heat shock protein family, is essential for the proper folding and assembly of newly introduced proteins in mitochondria. Recent studies by Zhang et al. [37] observed that HSPD1 expression was significantly lower in invasive pituitary adenomas compared to non-invasive ones, suggesting that downregulation of HSPD1 may contribute to tumor invasiveness. Further research by Zhan et al. [46] utilized proteomic data to analyze the signaling pathway network in human pituitary adenomas, revealing significant upregulation of components of certain mitochondrial complexes, such as ATP Synthase Subunit beta, Cytochrome C Oxidase Subunit 6B1, and nicotinamide adenine dinucleotide (NADH) dehydrogenase Fe-S protein 8, while glutathione peroxidase 4 was markedly downregulated. This study linked mitochondrial dysfunction and oxidative stress with cell cycle dysregulation in the pathogenesis of pituitary adenomas. Li et al. discussed the impact of mitochondrial dysfunction and dynamics on the development of pituitary adenomas, noting the dysfunction of respiratory complex I and decreased levels of lactate and lactate dehydrogenase A in pituitary oncocytomas [47]. The authors emphasized the critical role of mitochondrial proteins in regulating signaling pathways and promoting the progression of pituitary adenomas.

These findings underscore the significance of mitochondrial signaling pathway dysregulation in pituitary adenomas. Further research is essential to elucidate the specific mechanisms by which these proteins and pathways contribute to tumor development and to identify potential therapeutic targets for treating pituitary adenomas.

### 3.2. Oxidative Stress in Pituitary Adenomas

Oxidative stress refers to the imbalance between the production of ROS and the body’s ability to detoxify these reactive intermediates or repair the resulting damage. Mitochondrial oxidative stress is particularly crucial, as mitochondria are the primary sites of energy metabolism and are highly susceptible to oxidative damage due to their role in ROS production. Research as early as 1999 demonstrated, through both in vitro and in vivo models, that apoptotic cells exhibited significantly higher oxidative damage to mtDNA and increased glutathione oxidation compared to control cells. This study established a direct relationship between mitochondrial DNA damage and glutathione oxidation, underscoring the role of mitochondrial oxidative stress in apoptosis [48]. Several genetic disorders, including Down syndrome, Fanconi anemia, and Werner’s syndrome, have been linked to mitochondrial dysfunction and oxidative stress [49]. In the field of neurology, a growing body of research has highlighted the contributions of mitochondrial dysfunction and oxidative stress to the pathogenesis of various neurological disorders, such as epilepsy, neurodegenerative diseases, and ocular conditions. For instance, in epilepsy—a neurological disorder characterized by recurrent seizures—evidence suggests that oxidative stress plays a critical role in neuronal death [50]. Additionally, mitochondrial diseases associated with defects in oxidative phosphorylation are frequently linked to seizures, further reinforcing the connection between mitochondrial dysfunction and neurological disorders.

Oxidative stress and oxidative damage are common pathological and physiological features observed in pituitary adenomas, as validated in both cellular and animal studies. Nuclear factor erythroid 2-related factor 2 (Nrf2) serves as the primary regulator of the cellular antioxidant response, making its role in the oxidative stress response signaling pathways of pituitary adenomas particularly significant. Kelch-like ECH-associated protein 1 (KEAP1), an important antioxidant protein and the main regulatory factor of Nrf2, interacts with Nrf2 to induce an anti-stress response [51]. Increased levels of ROS activate the Nrf2–KEAP1 complex in the cytoplasm through various signaling pathways. Previous studies have shown that the expression of Nrf2 and phosphorylated Nrf2 is elevated in pituitary adenomas [52], along with the downstream effector HO-1, indicating the activation of the Nrf2 signaling pathway and its potential role in the development of pituitary adenoma cells [53]. Moreover, mitochondrial dysfunction-induced ROS increases have been confirmed in pituitary neuroendocrine tumors. A study employing the cytokinesis-blocking micronucleus cytometer found a significant increase in biomarkers related to chromosome and DNA damage in patients with pituitary adenomas, suggesting a correlation between chromosome/oxidative DNA damage and pituitary adenoma invasion [54]. One of the primary markers of oxidative DNA damage is 8-hydroxy-2′-deoxyguanosine (8-OHdG), whose elevated levels serve as established indicators of oxidative stress and cancer. Measurements of 8-OHdG levels revealed significantly higher concentrations in patients with prolactinoma and active acromegaly compared to controls [55,56]. Conversely, in patients with non-functional pituitary adenomas, researchers observed significantly lower levels of 8-OHdG relative to controls, suggesting that these patients may experience oxidative DNA damage, some of which could be repaired [57]. This finding underscores the association between oxidative DNA damage and pituitary adenomas. Furthermore, Zhang et al. utilized proteomics and metabolomics to analyze serum metabolic changes in male patients with pituitary insufficiency, aiming to elucidate the relationship between growth hormone deficiency and non-alcoholic fatty liver disease in these individuals. The analysis revealed significantly increased levels of biomarkers associated with mitochondrial dysfunction and oxidative stress, such as alanine, lactate, and creatine, in hypopituitarism compared to age-matched controls [58].

Understanding the mechanisms underlying mitochondrial oxidative stress and its role in the progression of pituitary adenomas is essential for developing effective treatment strategies. Investigating this area can help identify potential biomarkers for diagnosing and treating pituitary adenomas, ultimately facilitating the development of targeted therapies.

### 3.3. Mitochondrial Dynamics in Pituitary Adenomas

Mitochondria play a vital role in cancer biology, with their dynamics increasingly recognized as significant contributors to tumor growth and metastasis. The interplay between mitochondrial dynamics and cancer progression is complex and involves various molecular mechanisms. For example, the dysregulation of mitochondrial fusion proteins, such as mitochondrial dynamics protein of 49 kDa (MiD49), has been linked to the growth and metastasis of human pancreatic cancer. Specifically, the downregulation of MiD49 is associated with increased cell growth and metastatic potential in pancreatic cancer cells [59]. In lung adenocarcinoma, particularly interesting new cysteine–histidine-rich protein (PINCH-1) has emerged as a key regulatory factor in mitochondrial dynamics. Research has elucidated a signaling axis involving PINCH-1, dynamin-related protein 1 (DRP1), and pyrroline-5-carboxylate reductase 1, which regulates mitochondrial dynamics and proline synthesis, thereby promoting tumor growth [60]. Furthermore, findings from Tang et al. underscore the importance of mitochondrial dynamics in cancer, revealing that the induction of mitochondrial fission is crucial for maintaining liver cancer-initiating cells [61]. Overexpression of the mitochondrial fission factor enhances mitochondrial division, increasing stemness and the tumor initiation capacity of non-liver-cancer-initiating cells. These studies highlight the critical role of mitochondrial dynamics in carcinogenesis and suggest potential therapeutic targets for cancer treatment.

A key aspect of mitochondrial function in the progression of pituitary adenomas is mitochondrial dynamics, particularly the processes of division and fusion. Dysregulation of these dynamics, especially a reduction in mitochondrial division, has been associated with the invasiveness and high proliferation rates of growth hormone-secreting pituitary adenomas (GHPAs). Zhang et al. conducted a study comparing the mitochondrial morphology and dynamics in invasive GHPAs (IGHPAs) versus non-invasive GHPAs [62]. Their results demonstrated significant reductions in the number, volume, and membrane area of mitochondria in IGHPAs. Additionally, mRNA and protein levels of the key regulatory factor for mitochondrial division, DRP1, were found to be downregulated in IGHPAs, indicating mitochondrial hypodivision in these tumors. Mechanistically, DRP1-induced mitochondrial hypodivision can activate signal transducer and activator of transcription 3 (STAT3), leading to increased invasion and proliferation of GHPA cells. Moreover, dysregulation of mitofusin-1 expression or function can contribute to mitochondrial dysfunction, potentially facilitating the development and progression of pituitary adenomas. Other proteins involved in mitochondrial dynamics, such as mitofusin-2 and optic atrophy 1 (OPA1), also regulate hormone signaling pathways and tumor growth [63]. Research indicates that OPA1 expression is elevated in pituitary adenomas, resulting in alterations in mitochondrial elongation and function [64]. Furthermore, larger mitochondria were observed in pituitary adenomas characterized by increased fusion, suggesting a dysregulation of mitochondrial dynamics that may promote tumor growth and progression [47]. Overall, the interplay between mitochondrial division and fusion processes is crucial to the pathophysiology of pituitary adenomas. Further research is needed to fully elucidate the mechanisms by which these processes contribute to the development and progression of pituitary adenomas, paving the way for new therapeutic approaches.

## 4. Mitochondrial-Targeted Therapy in the Treatment of Pituitary Adenomas

The treatment of pituitary adenomas varies based on the type of adenoma and its associated symptoms. Currently, the primary treatment modalities include surgery, radiation therapy, and pharmacotherapy. Surgical treatment remains the cornerstone of pituitary adenoma treatment, except prolactinoma; for all other pituitary adenomas, the initial treatment is usually surgical resection [65,66]. With advancements in neuroimaging, endoscopic trans-sphenoidal surgery has emerged as the standard technique for managing pituitary adenomas [66]. However, surgery carries inherent risks, including potential damage to adjacent structures and challenges in completely excising the adenoma [67]. Radiation therapy is an alternative treatment option for tumors that are not suitable for surgical resection, recurrence, and residue [68]. Nonetheless, it is important to acknowledge potential complications, including the risk of developing secondary malignancies, such as osteosarcoma, following radiation therapy [69]. When surgery and radiation therapy fail to achieve biochemical remission and tumor control, drug therapy becomes essential for managing functional pituitary adenomas. Cabergoline, a dopamine agonist, is commonly used to treat prolactinomas and has been shown to effectively normalize prolactin levels and reduce tumor size in a significant proportion of patients [70]. In summary, while multiple treatment options exist for pituitary adenomas, each method has its limitations. To address these challenges and cater to individual patient needs, further research is essential to explore new treatment modalities and targets, aiming to enhance comprehensive care and improve patient outcomes.

Mitochondria play a vital role in cellular metabolism and energy production, positioning themselves as promising targets for therapeutic interventions across various diseases. Mitochondrial-targeted therapies in cancer treatment primarily focus on regulating cell proliferation and apoptosis. Nanosystems designed to deliver therapeutic agents directly to mitochondria represent a potential strategy for cancer treatment by disrupting energy metabolism, modulating ROS, and inducing mitochondrial autophagy through multiple pathways [71]. Furthermore, the processes of mitochondrial division, fusion, and autophagy are essential for protecting neurons from ischemic damage [72]. This underscores the potential of neuroprotective drugs aimed at mitochondrial dysfunction to enhance neuronal survival and function. Additionally, effective mitochondrial-targeted gene delivery systems have been developed to address mitochondrial DNA mutations and hereditary mitochondrial diseases. This represents a novel approach to gene therapy, aimed at correcting mtDNA mutations and improving mitochondrial function [73].

Research has demonstrated that mitochondrial dysfunction significantly contributes to the pathogenesis of pituitary adenomas. Specific pathways and drugs targeting mitochondria have emerged as potential therapeutic strategies for these tumors. For instance, melatonin and its inhibitors have been identified as mitochondrial-targeting agents that may be effective in treating pituitary adenomas [47]. Moreover, the *FGFR4* polymorphism allele has been shown to regulate the phosphorylation status of mitochondrial STAT3, resulting in altered hormone regulation and increased cell proliferation in pituitary cells. This suggests that targeting mitochondrial function could provide a novel approach for using somatostatin analogues in the management of pituitary adenomas [74]. Oxidative stress is also linked to the development of pituitary adenomas, and research indicates that targeting mitochondrial oxidative stress may serve as a viable treatment strategy for pituitary neuroendocrine tumors [63]. Mitoquinol mesylate, a mitochondrial-targeted antioxidant, has been shown to selectively induce protective autophagy, aiding in the elimination of damaged mitochondria and promoting cell survival [75]. Given the close relationship between oxidative stress and mitochondrial dysfunction in pituitary neuroendocrine tumors, addressing mitochondrial oxidative stress presents a promising therapeutic avenue.

In addition to targeting mitochondrial dysfunction, other pathways and mechanisms have been investigated as potential therapeutic targets for pituitary adenomas. Notably, the interaction between hypoxia-inducible factors (HIFs) and mitochondrial dysfunction has been identified as a potential target for treating solid tumors. Inhibiting tumor angiogenesis via the hypoxia-inducible factor 1-alpha/vascular endothelial growth factor/vascular endothelial growth factor receptor 2 signaling pathway is considered a promising strategy for targeted therapy in solid tumors, including pituitary adenomas [76]. By focusing on the interaction between HIFs and mitochondrial function, researchers hope to develop more effective treatments for these tumors. Furthermore, studies on mitochondrial dynamics have revealed that Drp1-mediated mitochondrial dysfunction may inhibit pituitary adenoma growth. Targeting Drp1 to inhibit excessive mitochondrial division could serve as a therapeutic strategy to control tumor growth in pituitary adenomas [77]. Mitochondrial autophagy, or mitophagy, is crucial for maintaining cellular health by selectively identifying and eliminating dysfunctional mitochondria. Numerous studies have highlighted the essential role of mitochondrial autophagy in diseases related to endocrine hormone imbalances, suggesting that targeted autophagy may enhance treatment outcomes for pituitary adenomas [78].

In summary, addressing mitochondrial dysfunction, oxidative stress, specific signaling pathways, and mitochondrial dynamics presents promising opportunities for developing more effective and targeted treatments for pituitary adenomas. However, additional research is essential to better understand the mechanisms underlying mitochondrial dysfunction in these tumors, which will help identify new therapeutic targets and enhance patient outcomes.

## 5. Natural Compound Therapy for Pituitary Adenomas

Natural compounds have garnered considerable attention in cancer treatment due to their potential therapeutic benefits. For example, histamine, an organic nitrogen-containing compound, has been shown to reduce lung metastasis formation in mice injected with melanoma cells, highlighting its potential as a tumor therapeutic agent [79]. Virk Baker et al. discussed the significance of phytoestrogens as adjunctive therapies for cancer, suggesting that natural compounds may enhance the efficacy of conventional chemotherapy [80]. Additionally, Wang et al. emphasized that natural product-induced cellular autophagy can inhibit tumor growth, underscoring the role of these compounds in regulating autophagy in tumor cells [81]. Collectively, these studies indicate that natural compounds have diverse and significant roles in cancer treatment, showcasing their promising potential for therapeutic applications.

Mitochondrial function plays a critical role in the treatment of pituitary adenomas, and natural compounds show promise by targeting cellular metabolism. Curcumin, the active ingredient in turmeric, has demonstrated anti-tumor effects across various cancer types. Research indicates that curcumin [82] not only inhibits cell proliferation in pituitary tumor cell lines but also regulates hormone secretion. In vivo studies have shown that curcumin effectively inhibits the growth of rat pituitary tumor cells (GH3), tumors in nude mice. Furthermore, investigations related to Cushing’s disease have revealed that curcumin reduces nuclear factor kappa-B activity, leading to decreased proliferation of AtT20 cells [83]. Additionally, curcumin downregulates B-cell lymphoma-extra large expression, resulting in mitochondrial membrane depolarization and the induction of apoptosis, further underscoring its potential therapeutic value in treating pituitary adenomas. The 18β-glycyrrhetinic acid (18β-GA), derived from licorice, exhibits significant cytotoxicity against pituitary adenoma cells and markedly inhibits tumor growth in nude mice [84]. Studies have shown that the anti-tumor effects of 18β-GA are associated with mitochondrial dysfunction and the mitogen-activated protein kinase (MAPK) pathways. Specifically, 18β-GA induces apoptosis in pituitary adenoma cells by activating the mitochondrial-mediated ROS/MAPK pathway [85]. Grifolic acid, a natural compound extracted from mushrooms, has been found to induce cell death in GH3 adenoma cells by inhibiting ATP production, likely through the suppression of NADH production [86]. Matrine, an alkaloid derived from the roots of Sophora flavescens, inhibits the proliferation of pituitary adenoma cells by promoting the nuclear localization of forkhead box O3A (Foxo3a) through the inhibition of the Protein Kinase B (Akt)/Foxo3a signaling pathway. This increase in nuclear Foxo3a levels activates downstream apoptotic gene expression. Experimental results indicate that matrine upregulates pro-apoptotic proteins Bim and Bax while downregulating the anti-apoptotic protein B-cell lymphoma-2, thereby promoting apoptosis [87].

Overall, there is a growing interest in harnessing natural compounds to target mitochondrial function in the treatment of pituitary adenomas. These compounds demonstrate promising anti-tumor activities and possess the ability to regulate cellular metabolism, positioning them as valuable candidates for the management of pituitary adenomas.

## 6. Conclusions and Prospects

A pituitary adenoma is an intracranial tumor that arises in the pituitary gland. Although most pituitary adenomas are benign, they pose significant clinical challenges, including treatment difficulties and uncertain prognoses, which can severely impact a patient’s quality of life. Mitochondria play a crucial role in cellular metabolism, energy production, and the response to various therapies. Research has shown a strong link between mitochondrial dysfunction and the development or progression of pituitary adenomas.

**Figure 1:**
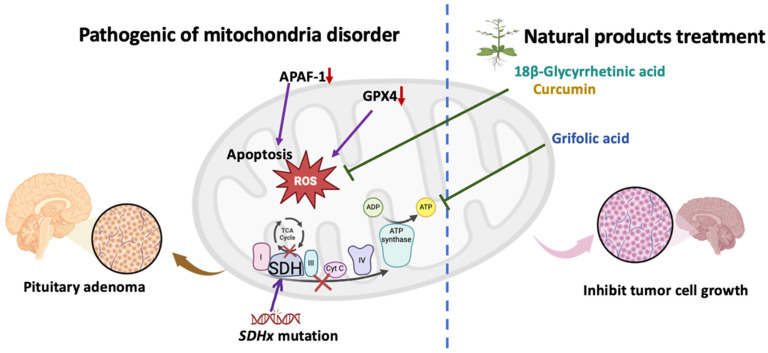
Schematic representation of mitochondrial dysfunction and intervention in pituitary adenomas. Succinate dehydrogenase (*SDH*) mutations disrupt the tricarboxylic acid (TCA) cycle and increase reactive oxygen species (ROS) levels, potentially driving the development of pituitary adenomas. Additional pathogenic mechanisms include apoptotic protease activating factor-1 (APAF-1) downregulation, glutathione peroxidase 4 (GPX4) inhibition, and enhanced apoptosis. Certain natural compounds, such as 18β-glycyrrhetinic acid, curcumin, and grifolic acid, may inhibit ROS production and promote adenosine triphosphate (ATP) synthesis, thereby reducing tumor cell growth and showing potential therapeutic effects against pituitary adenomas.

Mutations in mitochondria-related genes, such as *SDHB*, have been identified in pituitary adenomas, highlighting the significance of genetic and epigenetic changes in their pathogenesis. Mitochondrial dysfunction—which encompasses pathway networks, oxidative stress, and mitochondrial dynamics—has been recognized as a key factor in the development and progression of these tumors (Figure 1). Understanding the molecular mechanisms underlying the onset of pituitary adenomas is crucial for developing targeted therapies and improving patient prognoses. Given the complex relationship between mitochondrial function and pituitary adenomas, mitochondrial-targeted therapy has emerged as a promising approach for treatment, potentially leading to more effective and personalized strategies for patients facing these challenging conditions. Natural compounds have been reported to disrupt biological systems in various in vitro and in vivo disease models [88], demonstrating significant application value in both chemical biology and medicine [89]. In this review, we summarize natural compounds with therapeutic potential for treating pituitary adenomas, emphasizing that substances such as curcumin, grifolic acid, glycyrrhetinic acid, and matrine can modulate mitochondrial bioenergetics in different ways, thereby preventing mitochondrial oxidative stress-induced cell proliferation (Figure 1).

Moving forward, it is both necessary and challenging to gain novel insights into the treatment of pituitary adenomas from the perspective of mitochondrial involvement. Future research should aim to elucidate the role of mitochondria in the pathogenesis of these tumors and their influence on tumor progression. By identifying key mitochondrial targets, treatment strategies can be optimized, ultimately improving patient prognoses and contributing to better health outcomes.

## Figures and Tables

**Table 1:** *SDHx* defects in pituitary adenomas.

Gene	Genetic Variants	Pituitary Phenotype	References
*SDHA*	c.1873C>T, p.His625Tyr	Non-functioning pituitary macroadenoma	[38]
*SDHB*	c.343C>T, p.Arg115*	Aggressive cabergoline-resistant giant macroprolactinoma	[39]
*SDHB*	c.166-170delCCTCA	Pituitary adenomas (PAs) associated with pheochromocytomas/paragangliomas (Pheos/PGLs)	[40]
*SDHB*	c.487T>C, p.Ser163Pro	PA with adrenocorticotropic hormone (ACTH)	[33]
*SDHC*	c.20+74A>G	Pituitary adenoma	[41]
*SDHD*	c.298-301delACTC	Acromegaly	[42]
*SDHD*	c.242C>T, p.Pro81Leu	Giant prolactinoma	[33]
*SDHD*	c.149A>G, p.His50Arg	Non secretory PA patients	[33]
*SDHD*	c.53C>T, p.Ala18Val	PA with adrenocorticotropic hormone (ACTH)	[33]
